# The pathogenesis of iodide mumps: A case report

**DOI:** 10.1097/MD.0000000000008881

**Published:** 2017-11-27

**Authors:** Guilian Zhang, Tao Li, Heying Wang, Jiao Liu

**Affiliations:** Department of Neurology, the Second Affiliated Hospital, Medical School of Xi’an Jiaotong University, Xi’an, PR China.

**Keywords:** iodide mumps, iodide-containing contrast, MRI-DWI

## Abstract

**Relation:**

Iodide mumps is an uncommon condition, induced by iodide-containing contrast, and is characterized by a rapid, painless enlargement of the bilateral or unilateral salivary gland. At present, the pathogenesis of iodide mumps is not yet clear. It may be related to an idiosyncratic reaction, a toxic accumulation of iodine in the gland duct, or renal function damage leading to an iodine excretion disorder. This paper reports the clinical manifestations and magnetic resonance imaging results of one case of iodide mumps, which occurred after digital subtraction angiography.

**Patient concerns:**

A 66-year-old Chinese man presented to our department with a 1-month speech barrier and 1 day of vomiting. He had the history of high blood sugar, the history of high blood pressure and the history of Vitiligo. He had no history of allergies and had never previously received iodide-containing contrast. His renal function and other laboratory examinations were normal. During the digital subtraction angiography (DSA), the patient received approximately 130 mL of nonionic contrast agent (iodixanol). Five hours postsurgery, the patient experienced bilateral parotid enlargement with no other discomfort, such as pain, fever, skin redness, itching, hives, nausea, vomiting, or respiratory abnormalities.

**Diagnoses:**

We thought the diagnosis was iodide mumps.

**Intervention:**

Intravenous dexamethasone (5 mg) was administered.

**Outcome:**

20 hours post-DSA, after which the bilateral parotid shrunk. By 4 days postsurgery, the patient's bilateral parotid had recovered completely.

**Lessons:**

We found no obvious abnormal sequence signal in diffusion magnetic resonance imaging or the corresponding apparent diffusion coefficient. Our findings suggest that vasogenic edema may play an important role in the pathogenesis of iodide mumps.

## 1 Introduction

As modifications have refined the iodine contrast agent water solubility, ionic type, osmotic pressure, and viscosity, adverse reactions to these agents have decreased and the safety of these agents has increased. However, heterogeneity among individuals has caused some rare adverse reactions, such as iodide mumps. To date, less than 40 iodide mumps cases have been reported worldwide. We previously reported the first cases of bilateral mandibular gland inflammation in mainland China, which occurred after ioversol use.^[1]^ Although the pathogenesis of iodide mumps is not clear, an idiosyncratic reaction, a toxic accumulation of iodine in the gland duct, or renal function damage leading to an iodine excretion disorder may be involved in this process.^[1]^ However, the reported iodine mumps patients have not had personal/family histories of allergic diseases, and most had normal renal function, although diffusion-weighted magnetic resonance imaging (DWI) of the disease has not been reported. This article describes the clinical manifestations and the results of magnetic resonance imaging (MRI) examination after digital subtraction angiography (DSA) of one case and discusses the pathogenesis of iodide mumps.

## 2 Case report

**Figure 1:**
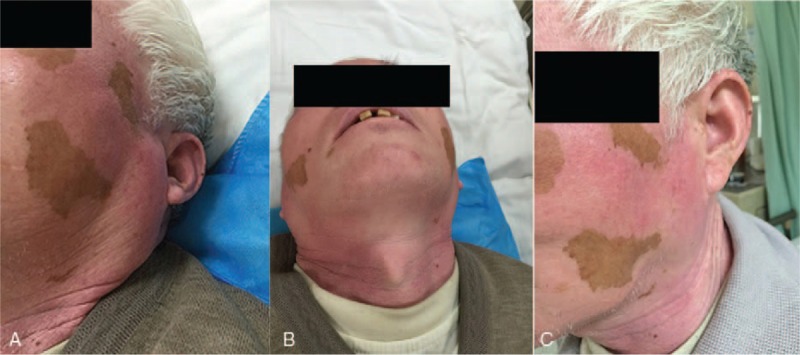
Images of the patient's parotid. Images of the parotid at 6 hours after symptom onset (A, B) and 20 hours after onset (C).

**Figure 2:**
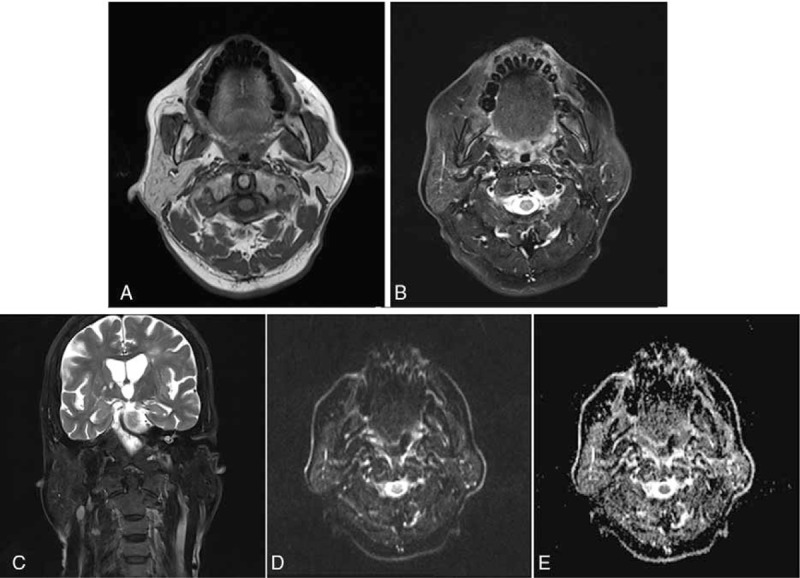
Parotid MRI and DWI. Representative MRI images showing the T1WI axial (A), T2WI axial (B), and T2WI coronal views (C). DWI (D) and ADC (E) images. ADC = apparent diffusion coefficient, DWI = diffusion-weighted magnetic resonance imaging, MRI = magnetic resonance imaging.

A 66-year-old Chinese man presented to our department with a 1-month speech barrier and 1 day of vomiting. He had a 1-month history of high blood sugar, poorly managed through diet control, and a 4-year history of high blood pressure, with a peak systolic blood pressure of 190 mm Hg. He was taking 300 mg of irbesartan once per day but had poor blood pressure control. The patient also had a 30-year history of drug therapy with Vitiligo. He had no history of allergies and had never previously received iodide-containing contrast. Physical examination showed slightly vague speech, left facial hypalgesia, grade V− right limb muscle strength, and a low right angulus oris, hypalgesic right limb, and Hoffmann sign-positive right side. Homocysteine was 37.80 μmol/L, and normal renal function (urea, creatinine, cystatin C). Results from other laboratory examinations were normal, including a routine blood analysis, a routine urinalysis, and tests for liver function, blood glucose concentration, blood lipid concentration, blood coagulation function, hepatitis B virus-specific antibody/antigen, anti-hepatitis C virus antibody, anti-hepatitis E virus IgM, human immunodeficiency virus-specific antibody/antigen, and anti-*Treponema pallidum* antibody. During the DSA, the patient received approximately 130 mL of nonionic contrast agent (iodixanol). Five hours postsurgery, the patient experienced bilateral parotid enlargement with no other discomfort, such as pain, fever, skin redness, itching, hives, nausea, vomiting, or respiratory abnormalities. Physical examination showed bilateral parotid area enlargement centred on the earlobe; the left side was 3′4 cm large, the right side was 3′3 cm large, and the masses were moderately firm with no obvious fluctuation and without vascular murmur (Fig. 1A and B). Bilateral parotid ultrasound showed a diffuse enlargement of the glands with a homogeneous echo. DWI indicated no obvious limited diffusion (Fig. 2). Intravenous dexamethasone (5 mg) was administered 20 hours post-DSA, after which the bilateral parotid shrunk (Fig. 1C). By 4 days postsurgery, the patient's bilateral parotid had recovered completely.

## 3 Discussion

Iodixanol is a nonionic iodine dimer-containing contrast media that is favored for its iso-molar properties. It contains mainly organic iodine and a tiny amount of inorganic iodide (1 mL of iodixanol contains ∼25–60 μg of iodine ions). With normal renal function, 97% of iodixanol is excreted unchanged in urine within 24 hours. Iodide becomes concentrated not only in the thyroid gland but also in other sites, such as the salivary glands. Salivary iodine is almost entirely in the inorganic form. The salivary iodide concentration is proportional to the plasma inorganic iodine (PII) concentration at physiological levels, and at PII concentrations of up to 100 μg/100 mL, there is a positive linear correlation between the two. Additionally, the salivary iodide concentration is inversely related to flow rate: as the flow rate rises, the salivary iodide concentration falls. At parotid salivary flow rates greater than 1 mL/min, however, the concentration remains constant. Furthermore, plasma iodide and perchlorate ions inhibit the iodide concentration in the salivary glands,^[2]^ which depends on the sodium–iodide symporter.^[3]^ When iodixanol is administered quickly by intravenous injection, it immediately reaches a peak level in the blood and the iodide is concentrated in the salivary gland. For susceptible patients, these excessive iodine ions are likely to cause an allergic or toxic reaction of the parotid gland. Therefore, the increased serum concentration of iodine may be related to the pathogenesis of iodide mumps.^[4]^

Animal and clinical studies have shown that nonionic dimer contrast agent can cause peripheral vasomotor dysfunction, which is not only the result of endothelial cell injury but also of the accompanying structural damage. For example, iodixanol can stimulate the Na^+^–K^+^ ATPase pump, which hyperpolarizes the vascular smooth muscle and hence diminishes Ca^2+^ influx. Additionally, iodixanol may cause relaxation by lowering the Ca^2+^ concentration as a consequence of altered Na^+^–Ca^2+^ exchange, resulting in vasodilation.^[5]^ It can also cause endothelial cell injury, increase vascular permeability via oxygen free radical formation, reduce nitric oxide production, and enhance the angiotensin (ANG) II reaction, leading to vascular edema.^[6,7]^

Our case had a sudden onset of parotid gland enlargement with no pain, fever, or other allergic manifestations, and an ultrasound showed a parotid homogeneity change, allowing us to eliminate local acute bleeding, abscess, and inflammatory swelling as the likely causes. Gilgen-Anner et al^[8]^ used histological analysis, skin tests, controlled re-exposure, premedication, and imaging studies to establish that salivary gland lesions in affected patients represented rare noninflammatory edema elicited by iodine. Additionally, Zhang et al^[1]^ found that the submandibular gland computed tomography(CT) image did not show obvious inflammation and edema, but the average CT density in both submandibular glands was lower than normal (20–40 HU), supporting the presence of noninflammatory edema.

MRI is sensitive to water balance changes^[9]^ and uses the signal intensity in proton images to show water proton diffusion. DWI is more focused on the Brownian motion of water protons within the tissue and is quantified by the apparent diffusion coefficient (ADC).^[10]^ The increase in signal intensity in DWI is due to the reduction of water protons within the tissue, which reflects the shift of water from the extracellular into the intracellular compartment, so the signal change is used to describe the inhibition of energy-dependent ion pumps and the breakdown of membrane potential.^[11]^ Changes in ADC values reflect the changes in extracellular and intracellular water levels and are related to cell energy metabolism and energy-consuming processes.^[9]^ In cytotoxic edema, the lesions observed in the T1- and T2-weighted images have no obvious signal intensity changes, but in DWI, because ATP-dependent ion conversion failure causes limited diffusion, the ADC value decreases.^[12,13]^ In vasogenic edema, due to capillary leakage and allergic reaction, and tissue edema, caused by extracellular water increased,^[11]^ the lesions producing low signals on T1-weighted and high signals on T2-weighted images become equisignal on DWI, with correspondingly increased ADC values.

The parotid gland is a glandular tissue rich in fat; under normal conditions, T1-weighted and T2-weighted images of this gland show slightly higher signals than the surrounding tissue. This study found that the patient's bilateral parotid enlargement did not significantly change the T1-weighted and T2-weighted images, had no limited diffusion detected by DWI, and had normal ADC values, suggesting that the edema is likely not inflammatory edema or cytotoxic edema. Based on to the sudden clinical manifestations of patients with iodine mumps, as well as their short symptom duration and complete recovery, we believe that patients with parotid gland edema may be experiencing toxic effects of iodine ions and that the allergic substances in the blood vessels lead to a subsequent increase in vascular permeability, resulting in vasogenic edema. However, DWI and the corresponding ADC sequences are not sensitive to these changes.

## 4 Conclusions

The pathogenesis of iodide mumps is not clear. Through this case, we found that the parotid gland edema induced by iodine contrast agent may be vasogenic edema, but its exact mechanism remains to be confirmed by further studies.

## Acknowledgments

Tao Li conceived and wrote the paper. Guilian Zhang reviewed and edited the manuscript. Katie Oakley, PhD, from Liwen Bianji, Edanz Group China (www.liwenbianji.cn/ac), for editing the English text of a draft of this manuscript. All authors read and approved the manuscript.
